# Efficacy of Composite Extract from Leaves and Fruits of Medicinal Plants Used in Traditional Diabetic Therapy against Oxidative Stress in Alloxan-Induced Diabetic Rats

**DOI:** 10.1155/2014/608590

**Published:** 2014-03-04

**Authors:** Brahm Kumar Tiwari, Dileep Kumar, A. B. Abidi, Syed Ibrahim Rizvi

**Affiliations:** ^1^Department of Biochemistry & Biochemical Engineering, Sam Higginbottom Institute of Agriculture, Technology and Sciences, Allahabad 211007, India; ^2^Department of Biochemistry, University of Allahabad, Allahabad 211002, India

## Abstract

Oxidative stress plays a vital role in diabetic complications. To suppress the oxidative stress mediated damage in diabetic pathophysiology, a special focus has been given on composite extract (CE) and making small dose of naturally occurring antidiabetic plants leaf and fruits. The aim of the present study was to evaluate the beneficial role of CE against alloxan- (ALX-) induced diabetes of Wistar strain rats. A dose-dependent study for CE (25, 50, and 100 mg/kg body weight) was carried out to find the effective dose of the composite compound in ALX-induced diabetic rats. ALX exposure elevated the blood glucose, plasma advanced oxidation product (AOPP), sialic acid demonstrating disturbed antioxidant status.CE at a dose of 100 mg/kg body weight restored/minimised these alterations towards normal values. In conclusion, small dose of CE possesses the capability of ameliorating the oxidative stress in ALX-induced diabetes and thus could be a promising approach in lessening diabetic complications.

## 1. Introduction

Diabetes mellitus is a syndrome characterized by chronic hyperglycemia and associated with absolute or relative deficiency in insulin secretion or insulin action [1]. Herbal medicine has been used as an antidiabetic therapy alone, along with insulin or other synthetic oral hypoglycemic agents. The use of synthetic agents is frequently associated with several undesirable side effects and fails to correct the fundamental biochemical lesion and diabetic complications [2]. The search for a cure for diabetes mellitus continues along with traditional and alternative medicine. Many herbal supplements have been used for the treatment of diabetes, but the scientific evidence to support their effectiveness has only been investigated for a few [3].

To suppress the oxidative stress mediated damage in diabetic pathophysiology, researchers usually look for naturally occurring antioxidants [4, 5]. Diabetes mellitus (DM) is strongly associated with oxidative stress [6]. Chronic hyperglycemia resulting from diabetes brings about a rise in oxidative stress due to overproduction of reactive oxygen species (ROS) as a result of glucose-autoxidation and protein glycosylation. Generation of ROS leads to oxidative damage of the structural components (such as lipids, DNA and proteins) of cells which culminate into complications affecting the eyes, kidney, nerves, and blood vessels [7]. Oxidative insult in cells is also created by the impairment in functioning of endogenous antioxidant enzymes because of nonenzymatic glycosylation and oxidation [8].

*Aegle marmelos* is a medium-sized deciduous tree found in dry forests and is also cultivated throughout India. Aqueous leaf extract of *Aegle marmelos* has been shown to improve the functional state of pancreatic cells in streptozotocin-induced diabetic rats. Antihyperglycaemic activity of *Aegle marmelos* is reported for leaf extract in glucose fed hyperglycaemic rats [9]. Oral administration of leaves of plant at 5 g/day significantly ameliorates blood glucose level in non insulin dependent diabetes mellitus patients [10]. *Azadirachta indica *is an evergreen tree which grows throughout India. Its leaf extract has been observed to produce antihyperglycaemic activity in streptozotocin diabetic rats without altered serum cortisol level [11]. The plant exerts its pharmacological activity independent of its time of administration, that is, either prior to or after streptozotocin administration [12]. *Murraya koenigii* leaves extract is considered extensively as antidiabetic agent and as a spice and condiment in India and other tropical countries. It is also found to be useful in the treatment of kidney infirmities [13]. Feeding different doses of *Murraya koenigii *leaves to diabetic rats plays a role in control of mild diabetes but in case of moderate, severe, and type I diabetes, this agent alone is not likely to be useful [14]. *Murraya koenigii* leaf extract significantly decreased the level of blood glucose in experimental diabetic rats [15].

*Ocimum sanctum *is an annual tropical herb grown all over India and used in household remediation [16]. Alcoholic extract of leaves significantly lowered the blood glucose in normal and alloxan diabetic rats [17]. *Syzygium cumini* is distributed throughout India and Indian folk medicine is replete with its use for the treatment of diabetes [11]. Ethanolic extract of seed kernels (200 mg/kg b.w.) has been observed to improve glucose tolerance [3]. Traditionally in Indian medicinal system, it is always found that composite extract is the most effective compared to single plant extract. The present study aims to study the potent antidiabetic activity of composite extract (CE) of *Aegle marmelos, Azadirachta indica, Murraya koenigii, Ocimum sanctum* leafs, and *Syzygium cumini* fruits in alloxan-induced diabetic rats comparing the effect with insulin.

## 2. Materials and Methods 

### 2.1. Chemicals

N-Acetylneuraminic acid (NANA), resorcinol, and chloramine-T were purchased from Sigma Aldrich, India. All other chemicals were of highest purity, available from Merck, India, and HIMEDIA Labs, India.

### 2.2. Plant Material

*Aegle marmelos*,* Azadirachta indica*,* Murraya koenigii*, and *Ocimum sanctum* were collected from Sam Higginbottom Institute of Agriculture, Technology & Sciences orchard, whereas *Syzygium cumini* fruits were collected from a local market in Allahabad.

### 2.3. Preparation of Composite Extract (CE)

The collected plants leaves and fruits were dried at room temperature, pulverized by a mechanical grinder, and sieved through 40 mesh. The fine powder of leaves and fruits was extracted with 80% methanol using a Soxhlet at boiling temperature (60°C) up to 10 h separately; a dark green coloured extract was obtained for *Aegle marmelos*,* Azadirachta indica*,* Murraya koenigii*, and *Ocimum sanctum* leaves whereas the extract obtained from *Syzygium cumini* fruits was dark brown in colour. The extract was then evaporated to dryness in a boiling water bath. The obtained extract was collected in air tight dark bottle separately and stored at 4°C until time of use. The extracts of all plants were diluted in water on the day of experiment and administered by oral gavage at dose of 25 mg, 50 mg, and 100 mg/kg body weight (all doses contain same concentration ratio of all extracts) in a fixed volume of 1 mL.

### 2.4. Experimental Induction of Diabetes in Rats

The rats were injected with alloxan monohydrate (2,4,5,6-tetraoxyprimidine) dissolved in sterile 0.9% normal saline at a dose of 150 mg/kg body weight intraperitoneally [18, 19]. Since experimentally alloxan is capable of inducing insulin dependent diabetes mellitus (IDDM) (which destroys pancreatic *β*-cells and induces type 1 diabetes), the rats were then kept for the next 24 h on 5% glucose solution bottles in their cage to prevent hypoglycaemia [20]. After one week, diabetic condition was confirmed through measurement of fasting blood glucose level. Glucose was measured using a glucometer (GlucoCare Ultima).

### 2.5. Animal Model and Study Protocol

The experiment was carried out with 36 male Wistar rats (4 ± 0.5 months old) with body weight in the range 150 ± 15 g. They were housed in a temperature controlled facility (25 ± 5°C) with 12 h light-dark cycle for at least 1 week. All rats were fed with a normal laboratory diet of nutrient rich pellets containing total energy as fat, protein and carbohydrates and had free access to drinking water. After the stabilization period of one week, the rats were randomly divided into six groups, containing six animals in each group (*n* = 6): group I: control: receiving no treatment/supplementation; group II: induced diabetes: rats were injected single dose intraperitoneally of alloxan [18, 19]; group III: diabetic insulin group was treated twice a day by subcutaneous injection of three units of NPH insulin (NPH huminsulin, Lilly Egypt) [21]; groups IV, V, and VI: rats were administered daily composite extract (25 mg/kg, 50 mg/kg, and 100 mg/kg body weight, resp.) via gavage technique (oral route) up to 35 days [22]. All treatments were carried out up to 35 days. The animals of the first group were simultaneously administered water until 35 days.

### 2.6. Collection of Blood and Isolation of Serum and Plasma

After the end of the treatment period, rats were sacrificed under light anaesthesia (chloroform). Blood was collected by cardiac puncture into 10 unit/mL heparin rinsed anticoagulant syringes (for the collection of serum no anticoagulant was used), and then red blood cells were pelleted by centrifugation at 800 g for 10 min at 4°C. Separated plasma and serum were immediately frozen at −80°C until use for biochemical assay. All protocols for experiments were approved by the Animal Care and Ethics Committee of University of Allahabad.

### 2.7. Assay of Advanced Oxidation Protein Products

Determination of AOPP levels was performed by modification of the method of Witko-Sarsat [23]. Two mL of plasma was diluted 1 : 5 in PBS: 0.1 mL of 1.16 M potassium iodide was then added to each tube, followed by 0.2 mL acetic acid after 2 min. The absorbance of the reaction mixture was immediately read at 340 nm against a blank containing 2 mL of PBS, 0.1 mL of KI, and 0.2 mL of acetic acid. The chloramine-T absorbance at 340 nm being linear within the range of 0 to 100 mmol/L, AOPP concentrations were expressed as *μ*mol·L^−1^ chloramine-T equivalents.

### 2.8. Determination of Plasma Sialic Acid (NANA) Level

It was performed by the method proposed by Spyridaki and Siskos [24]. To determine the sialic acid level in plasma, 0.10 mL of 0.04 M periodic acid was added to a glass tube containing 500 *μ*L diluted (20 times) sample solution. It was mixed thoroughly and allowed to stand in ice bath for 30 min. Thereafter, 1.25 mL of resorcinol working solution (5 mL of 6.0% resorcinol solution, 0.125 mL of 0.1 M copper sulphate solution, and 19.875 mL of distilled water, brought to a final volume of 50 mL with 10 M HCl) was added, mixed and heated at 98°C for 5 min. Tubes were cooled in an ice bath for approximately 2 min. Lastly 3.25 mL of n-butanol was added. The solutions were mixed, vigorously and the tubes were placed in a water bath at 37°C for 3 min for colour to stabilize. Immediately after removing the solutions from the water bath their absorbance was measured at 625 nm against a reagent blank. A calibration graph was prepared with standard solutions of NANA in the range 20–200 *μ*M and the unknown concentrations of total sialic acid in samples were calculated. Plasma sialic acid is measured as *μ*M.

### 2.9. Other Biochemical Analysis

Blood glucose level was measured by glucometer (GlucoCare Ultima), total cholesterol and triglycerides were estimated by enzymatic method, HDL was assayed by phosphotungstic acid method using reagent kit procedural guideline and detail (ERBA Diagnostics Mannheim, Germany). Creatinine was measured by modified Jaffe's reaction (Span Diagnostic Ltd., Surat, India), free fatty acid was measured by Duncombe [25], and alkaline phosphatase was assayed by method of Moss et al. [26].

### 2.10. Statistical Analysis

Statistical analyses were performed using GraphPad Prism version 5.00 for Windows, GraphPad Software, San Diego, California, USA. Results were expressed as the mean ± S.D. for statistical analysis of the data group means and were compared by one-way analysis of variance (ANOVA) followed by Tukey's multiple comparison Test. *P* < 0.05 was considered to be statistically significant.

## 3. Results

### 3.1. Blood Glucose Level

**Figure 1:**
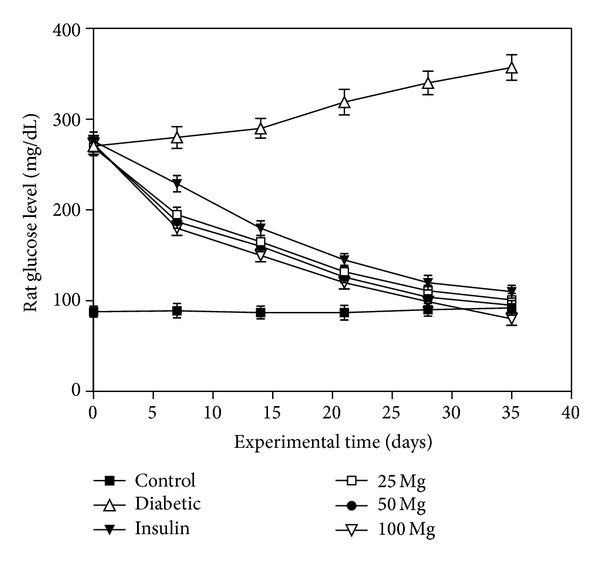
Levels of glucose in experimental rats after 1, 7, 14, 21, 28, and 35 days of treatment. Alloxan-induced diabetic rats have increased glucose level compared to normal control rats. Daily supplementation of CE dose (25, 50, and 100 mg/kg body weight) and subcutaneous injection of insulin significantly decreased the glucose level compared to diabetic rats. CE dose 100 mg/kg body weight showed significantly (*P* < 0.05) higher glucose lowering effect compared to insulin treated group. Glucose level in blood is expressed in mg/dL. Values indicate mean ± S.D. of six rats per group.

The dose-dependent effects of composite extract (CE) on blood glucose in normal and experimental rats are shown in Figure 1. The blood glucose was significantly elevated in diabetic rats as compared to normal control rats. Oral administration of CE at 25, 50, and 100 mg kg^−1^ body weight significantly lowered the blood glucose level as compared to untreated and insulin treated diabetic rats. CE at a dose of 100 mg kg^−1^ body weight restored the blood glucose to normal levels.

### 3.2. Advance Oxidation Protein Product

**Figure 2:**
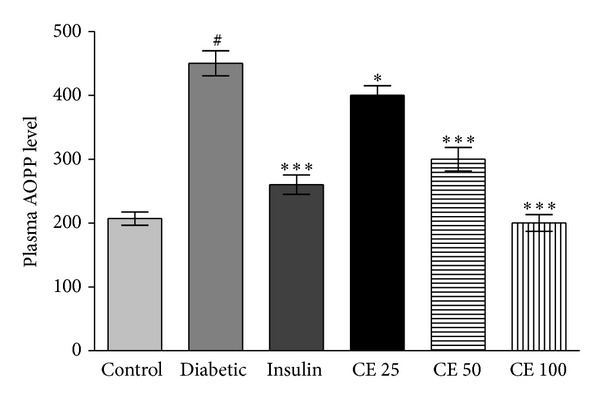
Advanced oxidation protein products (AOPP) level in normal, control, and experimental groups of Wistar rats. AOPP was significantly (^#^ *P* < 0.001) increased in alloxan-induced diabetic group of rats compared to control. Oral administration of CE dose 25, 50, and 100 mg/kg body weight and subcutaneous injection of insulin up to 35 days significantly (***P < 0.001 and *P < 0.05) decreased AOPP level compared to diabetic control group. AOPP level is expressed in *μ*mol·L^−1^ chloramine-T equivalents. Values are given as mean ± S.D. for 6 rats in each group.

The level of advance oxidation protein product (AOPP) in plasma was significantly increased in alloxan-induced diabetic rats when compared to normal control rats. Administration of CE offered significant reduction in the level of AOPP level in diabetic rats (Figure 2). CE at a dose of 100 mg kg^−1^ body weight tried to maintain AOPP plasma level near to the normal rats.

### 3.3. Plasma Sialic Acid Level

**Figure 3:**
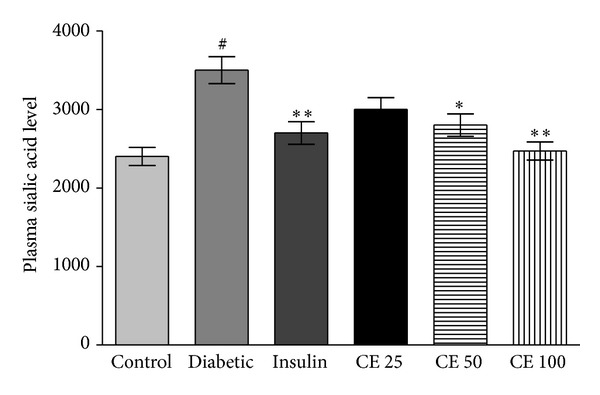
Plasma sialic acid content in normal and experimental groups. Alloxan-induced diabetes revealed significant (^#^ *P* < 0.001) increase in level of sialic acid compared to normal control groups. Oral administration CE dose (25, 50, and 100 mg/kg body weight) and subcutaneous injection of insulin up to 35 days significantly (*P < 0.05, **P < 0.01) decreased level of plasma sialic acid in alloxan-induced diabetic rats; therefore, CE 25 mg/kg was not significantly different to diabetic control rats. CE dose was not statistically significant with insulin treated group. Value is expressed in *μ*M. Values are expressed as mean ± S.D. for 6 rats in each group.

The extract showed a mild sialic acid lowering effect in nondiabetic rats, while the levels of the plasma sialic content were significantly increased in diabetic rats. The composite extract treated diabetic rats have shown a significant retrieval in the levels of sialic acid in the plasma level as shown in Figure 3 composite extract treated diabetic animals displayed significant reduction towards nondiabetic levels.

### 3.4. Serum Lipid Profile

**Figure 4:**
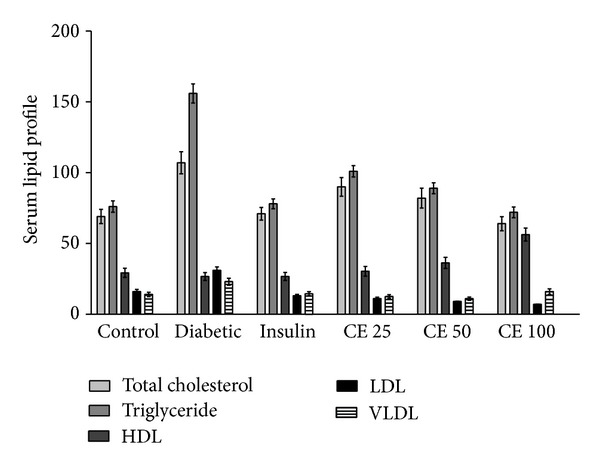
Serum lipid profile: total cholesterol, triglyceride, LDL, and VLDL significantly (*P* < 0.001) increased in alloxan-induced diabetic rats when compared with the normal control rats; however, HDL level was not significantly increased. Oral administration of CE offered significant (*P* < 0.05) decrease in total cholesterol, triglyceride, LDL, and VLDL and also significantly increased HDL level in diabetic rats.

The change in the level of serum lipid profile in control and experimental rats is illustrated in Figure 4. Except for HDL, total cholesterol, triglyceride, LDL, and VLDL significantly increased in alloxan-induced diabetic rats when compared with the normal control rats. Oral administration of CE offered significant decrease in total cholesterol, triglyceride, LDL, and VLDL and also increase in the HDL level in diabetic rats. CE at a dose of 100 mg kg^−1^ body weight and also insulin restored serum lipids approximately to the normal level but serum HDL level significantly increased despite CE treatment.

### 3.5. Serum Creatinine

**Figure 5:**
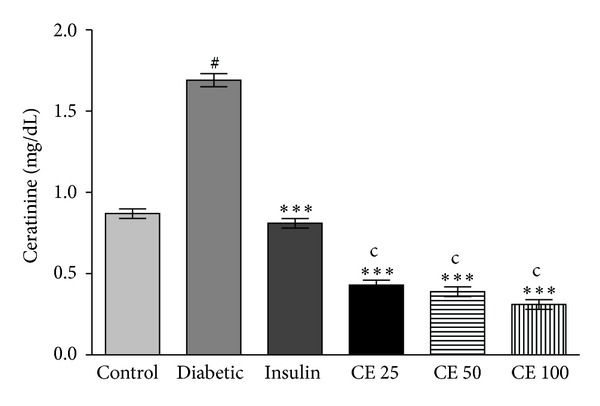
Serum creatinine level in CE treated and alloxan-induced diabetes rats. Creatinine level was significantly (^#^ *P* < 0.001) increased in alloxan-induced diabetic group compared to normal control group. Oral supplementation of CE doses 25, 50, and 100 mg/kg body weight and subcutaneous injection of insulin significantly (***P < 0.001) decreased creatinine level compared to diabetic control rats. CE doses 25, 50, and 100 mg/kg body weight showed significant (^c^ *P* < 0.001) difference between insulin treated groups. Creatinine concentration is expressed in mg/dL. All values are expressed as mean ± S.D. for 6 rats in each group.

The level of serum creatinine is shown in Figure 5. The level of creatinine was significantly higher in diabetic rats as compared to normal rats. The oral dose of CE 25, 50, and 100 mg kg^−1^ body weight decreases the creatinine level, respectively; the effect of CE was dose dependent.

### 3.6. Serum Free Fatty Acid

**Figure 6:**
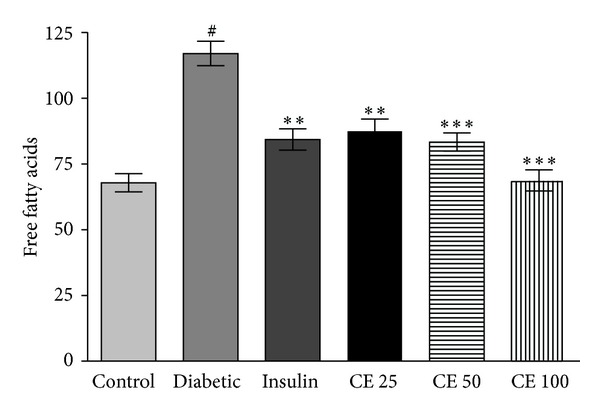
The level of serum free fatty acid was significantly (^#^ *P* < 0.05) higher in diabetic rats as compared to normal rats. Oral administration of CE doses 25, 50, and 100 mg/kg body weight and subcutaneous injection of insulin significantly (**P < 0.01, ***P < 0.001) lowered the serum free fatty acid as compared to untreated diabetic rats. CE 25, 50, and 100 showed better result but there was no significant difference with insulin treated group. Values of free fatty acid content expressed in *μ*M. All values are expressed as mean ± S.D. for 6 rats in each group.

The level of serum free fatty acid is shown in Figure 6. Serum free fatty acid was significantly higher in diabetic rats as compared to normal rats. Oral administration of CE lowered the serum free fatty acid as compared to untreated diabetic rats.

### 3.7. Serum Alkaline Phosphatase

**Figure 7:**
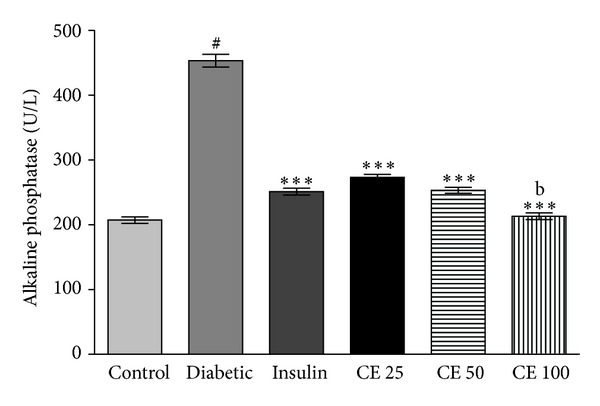
Serum marker of hepatic dysfunction in normal and CE treated rats. ALP activity significantly (^#^ *P* < 0.001) increased in diabetic rats compared to normal control rats. CE dose and insulin administration significantly (***P < 0.001) lowered the ALP activity in diabetic rats compared to diabetic control rats. CE dose 100 mg/kg showed significant (^b^ *P* < 0.01) difference between insulin treated groups. ALP activities expressed in IU/L. Values are expressed as mean ± S.D. for 6 rats in each group.

The level of alkaline phosphatase in serum was significantly increased in alloxan-induced diabetic rats when compared to normal control rats. Administration of CE offered significant reduction in the level of alkaline phosphatase in diabetic rats. CE at a dose of 100 mg kg^−1^ body weight restored the alkaline phosphatase level near to the normal level compared to normal rats (Figure 7).

## 4. Discussion

Many of the individual ingredients (used in the present study) of this polyherbal product have been shown to have hypoglycemic effect in earlier studies [3, 11]. Individually all plants used in the study contain medicinal ingredients which have been shown to elicit hypoglycaemic effect in diabetic rats, albeit in a large dose [9, 27]. Previous studies have revealed that the combined extract of *E. jambolana* (seeds) and *A. marmelos *(leaves) produces hypoglycaemic effect by increasing serum insulin levels and decreasing serum lipid levels; this effect is attributed to the synergistic effect of the combined plant extracts [28]. A study has also revealed that the *O. Sanctum* leaf extracts have stimulatory effects on physiological pathways of insulin secretion which may underlie its reported antidiabetic action [29]. In case of *Azadirachta indica* leaves the probable case of reduction of blood glucose level might be due to increased peripheral uptake of glucose and increased sensitivity of insulin receptors [30] and also in case of *M. Koenigii *the reduced blood glucose level could result due to delayed absorption of the glucose from the gastrointestinal tract [31]. Continuous treatment for 35 days with the CE (100 mg kg^−1^ body weight) caused a significant decrease in the blood glucose level of diabetic rats (Figure 1). This is an interesting observation, as the continuous use of the extract or the accidental overdose of CE will not result in hypoglycaemic shock.

Free radical oxidative damage leads to loss in specific protein function. Since proteins have varied biological functions, there are often unique functional consequences resulting from their modification. It is estimated that almost every third protein in a cell of older animals is dysfunctional as enzyme or structural protein due to oxidative damage [32]. Oxidative modulation of proteins due to reduced radical scavenging activity of plasma patients may be one of the reasons of altered physiological processes in type 2 diabetic patients [28]. Therefore, the measurement of the protein oxidation is a clinically important factor for the prediction of the diabetes or degree of oxidative stress in diabetes and stress-related diseases. Advanced protein oxidation products (AOPP) are defined as dityrosine containing crosslinked protein products due to action of chloraminated oxidants, mainly hypochlorous acid and chloramines, produced by myeloperoxidase in activated neutrophils. It is considered a reliable marker for estimating the degree of protein oxidative modification [23]. Oxidation of proteins can lead to a whole variety of amino acid modifications; it may be selective and specific. Accumulation of protein products is associated with a number of diseases, including amyotrophic lateral sclerosis, Alzheimer's disease, respiratory distress syndrome, muscular dystrophy, and rheumatoid arthritis [33]. A few recent studies have shown that the value of AOPP is modulated during aging in animals, providing an efficient additional correlation between oxidative stress and aging [34]. The present study showed that CE is as effective as insulin in decreasing the levels of AOPP value and blood glucose level in alloxan diabetic rats (Figure 2).

RBCs are highly susceptible to oxidative damage due to the high cell concentration of oxygen and haemoglobin, a powerful promoter of the oxidative process [35]. We present evidence (Figure 3) to show increased plasma sialic acid in diabetic rats; CE treatment restored sialic acid to normal level. The increased plasma sialic acid is also an indicator of oxidative stress during diabetic condition. It has recently been reported that plasma sialic acid level is increased during aging process which is strongly correlated with oxidative stress [36].

The level of serum lipids is usually elevated in diabetes which represents a high risk factor for coronary heart disease. Under normal condition, insulin activates the enzyme lipoprotein lipase, which hydrolyses triglycerides. However, in a diabetic state, lipoprotein lipase is not activated in sufficient amount due to insulin deficiency resulting in hypertriglyceridemia [28]. The treatment with CE doses 25 mg, 50 mg, and 100 mg/kg of body weight led to significant decrease in total cholesterol, triglyceride, LDL and VLDL levels and increase in the level of HDL (Figure 4). The high prevalence of vascular complications was associated with age, BMI, and triglyceride of diabetic patients. Effort to treat triglyceride appropriately among elderly diabetic patients could be considered as a prime target [37]. The regression of the diabetic state on CE administration increases the utilization of glucose, thereby depressing the mobilization of fat (Figure 6). Increased levels of these enzymes together with ALP and ACP are reported to be associated with liver dysfunction and leakage into blood stream in diabetes shown Figure 7 [38]. Negative nitrogen balance with enhanced tissue proteolysis and decreased protein synthesis can contribute to enhanced serum urea and creatinine levels (Figure 5), indicating impaired renal functions in diabetic animals [39]. The effect of CE at a dose of 100 mg kg^−1^ body weight restored all the deranged parameters in alloxan-induced rats to normal levels.

## 5. Conclusion

In conclusion we show that the CE is a viable treatment strategy for maintenance of diabetic condition including oxidative stress. In its antidiabetic effect, CE is better than a large single extract dose because the different active compounds in CE probably act synergistically to exert their action through different mechanisms providing better protection against diabetic condition and its associated complications.
